# The Incidence of Common Complications, Including Ectropion and Entropion, in Transconjunctival and Subciliary Approaches for Treatment of ZMC Fractures

**DOI:** 10.30476/DENTJODS.2020.84853.1101

**Published:** 2021-06

**Authors:** Momeni Roochi Mehrnoush, Jalal Abbasi Amir, Zahedipour Hamed, Hajiani Narges

**Affiliations:** 1 Fellowship of Maxillofacial Trauma, Dept. of Oral and Maxillofacial Surgery, Sina Hospital, Craniomaxillofacial Research Center, Shariati Hospital, Tehran University of Medical Sciences, Tehran, Iran; 2 Fellowship of Facial Cosmetic and Reconstructive Surgery, Dept. of Oral and Maxillofacial Surgery, Sina hospital, Craniomaxillofacial Research Center, Shariati Hospital, Tehran University of Medical Sciences, Tehran, Iran; 3 Dept. of Oral and Maxillofacial Surgery, Zahedan University of Medical Sciences, Zahedan, Iran; 4 Craniomaxillofacial Research Center, Shariati Hospital, Tehran University of Medical Sciences, Tehran, Iran; 5 Resident Dept. of Oral and Maxillofacial Surgery, Sina hospital, Craniomaxillofacial Research Center, Shariati Hospital, Tehran University of Medical Sciences, Tehran, Iran

**Keywords:** Zygomaticomaxillary Complex Fractures, Transconjunctival, Subciliary, Entropion, Ectropion

## Abstract

**Statement of the Problem:**

Treating zygomaticomaxillary complex fractures (ZMC Fx) can result in postoperative complications that should be minimized by choosing the best surgical approach.

**Purpose:**

This study compared incidence rates of some common postoperative complications with emphasis on ectropion (an outward curling of the lower eyelid) and entropion (an inward curling of the lower eyelid) occurring with transconjunctival or subciliary approaches for the treatment of ZMC fractures.

**Materials and Method:**

This prospective study enrolled 80 patients with ZMC Fx who had been surgically treated. Patients were visited within one month and five months postoperatively by the same surgeon. An information checklist was completed for each patient for clinical assessment of postoperative complications.

**Results:**

There was no significant difference between the two groups in the type of trauma (simple or comminuted) (*p*= 0.1) or the frequency of ectropion and entropion one month and five months postoperatively (*p*> 0.05). The same results were observed for history of massage under the eye or around the field of surgery (*p*= 0.151), scleral show (*p*= 0.414), history of post-surgical epiphora (overflow of tears and accumulation of tear) (*p*= 0.059), duration of the use of suspension/frost sutures (used to prevent eyelid distortion secondary to wound injury applied at the skin inferior to the incision to help elevate the lid) (*p*= 0.057), and the use of porex (an alloplastic material over the defect in the orbital floor) (*p*= 0.91).

**Conclusion:**

There was no significant difference between the transconjunctival approach and the subciliary approach in terms of common postoperative complications such as ectropion and entropion.

## Introduction

In recent years, assault, interpersonal violence, motor vehicle accidents (MVA), occupational accidents, and sports injuries have resulted in various physical injuries, including maxillofacial trauma [ 1]. The oral and maxillofacial area is one of the most important anatomical sites in terms of function and esthetics, and about one-third of all injured patients have some type of damage to this area [ 2]. As this area is one of the most vulnerable parts of the body, fractures in this area can cause serious complications and adverse outcomes for patients. They often cause degrees of deformity and impairment of function, which makes treatment challenging. Therefore, physicians have two types of responsibility toward their patients, which are to repair defects in a way that ultimately results in the relatively same facial appearance as before the trauma and to restore the initial function [ 3]. The treatment of maxillofacial injuries is very important because of the proximity of this area to vital organs, including the brain and the globe, and because of esthetic issues [ 4]. The results of fractures in this area differ according to the severity and cause of the trauma, which varies from country to country [ 5- 6]. Today, assault and physical quarrels are among the common causes of maxillofacial fractures [ 7] and often cause damage to soft tissue, tooth, and bone components [ 8]. Zygomaticomaxillary complex (ZMC) fracture (ZMC Fx) is the second most common facial fracture after nasal ones due to the prominent position of the zygoma in the facial skeleton. The frequency of these fractures is four times greater in men than in women, and they usually occur in the second and third decades of life. MVA and interpersonal violence are the most common causes of this type of trauma [ 9]. Bilateral ZMC fractures are rare and comprise about 4% of these fractures. ZMC Fx can disrupt the facial contour, disturb vision, limit ocular movement, and interfere with the function of the mandible [ 10 ]. The most common complications in the treatment of ZMC fractures are dehiscence, hematoma, and seroma, lymphedema, a shortening of the lower eyelid, ectropion, entropion, infraorbital nerve disorders, implant infection, diplopia, enophthalmos, blindness, and retrobulbar hemorrhage [ 9 ].

Ectropion is an outward curling of the lower eyelid with mild, moderate, and severe degrees. Mild ectropion is when the curling of the eyelid from the globe is low (grade 1), moderate ectropion is when the curling of the eyelid from the globe is associated with the vertical shortening of the lower eyelid (grade 2), and severe ectropion is when true eversion occurs (grade 3). Mild and moderate degrees heal with time and gentle massage, but severe ectropion needs to be treated surgically. Entropion is the inner curling of the lower eyelid; this condition is less prevalent but more worrisome than ectropion, because it can damage the globe by the eyelashes. Entropion differs in grade from 1 to 4; those with grade 1 have excellent anatomic and functional results, and those with grade 4 have poor outcomes [ 11 ]. All grades require corrective surgery. The incidence of ectropion or scleral show with the subciliary approach with skin-muscle dissection has been reported to range from 6% to 18% by different studies [ 12 ]. It has also been shown that ectropion is more likely to occur in older people [ 13 ]. However, in the study of Appling *et al*. [ 14 ], the incidence of scleral show from treatment using the transconjunctival approach was 3%, and no entropion was observed.

The purpose of the current study was to compare the incidence rates of complications, especially ectropion and entropion, occurring when the transconjunctival or the subciliary approach is used in treating fractures of the ZMC.

## Materials and Method

This prospective study included 80 patients with ZMC fracture who were surgically treated in Shariati and Sina hospitals in Tehran in 2017. The inclusion criterion was the presence of a pure ZMC Fx, and the exclusion criteria comprised the presence or history of trauma-resulted lid deformities, pan facial fractures, and previous lid laxity. All participants signed an informed consent agreement. The study was approved by the Tehran University of Medical Sciences Research Ethics Committee. The patients underwent surgery performed by a single surgeon using either the transconjunctival or the subciliary approach. The type of surgery was chosen randomly. Prior to surgery, to assess the elasticity of the lower lid, snap test and the distraction test were performed to ensure that all patients had normal lower lid tissue. The snap test is performed by pulling the lower lid downward and releasing it to evaluate how quickly it returns to its initial place; the distraction test is done to roll out the laxity of lid (if the lower lid pulls more than 7 mm from the globe, then laxity may be present). In this method, two types of incisions are described for the transconjunctival approach in terms of the relation between the orbital septum and the dissection path that includes preseptal and retroseptal incisions. In this study, the transconjunctival approach with retroseptal incision in association with lateral canthotomy was used, which has a better cosmetic advantage than other common methods. All patients in this group received a lateral canthotomy. At the end of surgery with this approach, closure of the conjunctiva was done using three catgut sutures. With the subciliary approach, the skin incision is 2 mm under the gray line of the lower eyelid and extends over the entire length of the eyelid. Incision may also extend 1 to 1.5cm in the crease below the lateral canthal ligament.

During the surgery, porex was used in 58% (22 people) of patients in the transconjunctival group and in 40% (17 people) of patients in the subciliary group. Not one orbital implant crossed over onto the orbital rim, and all were posterior to the anterior orbital axis.

Furthermore, suspension sutures were used during surgery for all patients in the transconjunctival group so that in this group, lateral canthus suspension and zygomaticus major suspension were utilized. In the subciliary group, however, only zygomaticus major suspension was used for all patients except three of them who required no suspension suture.

To compare the incidence rates of ectropion and entropion and some common complications between the transconjunctival and subciliary groups, patients were visited within one month and five months postoperatively by the surgeon. During these visits, the lower eyelid was precisely examined, and after the correct adjustment of the patient's head position, the frontal image was prepared and stored for comparison with the next visit. Patient status, in terms of incidence and degree of ectropion and entropion, was determined according to the existing categorization. All patients were thoroughly informed about the treatment method, and each of them signed a written consent form before participating in this study. For each patient, a checklist containing information such as age, sex, cause of trauma, type of fracture, surgical procedure, entropion, ectropion, and other complications after surgery was completed.

## Data analysis

The collected data was integrated into the SPSS-21 soft-ware and analyzed. Then, mean and standard deviation were used to describe the data and the chi-square test was used to compare the frequencies of ectropion and entropion in the two studied surgical approaches. The significance level in this study was considered as *p*<0.05.

## Results

From 80 patients with fractures, 38 patients underwent surgery with the transconjunctival approach with a mean of 29.63 days between trauma and surgery, and 42 patients underwent surgery with the subciliary approach with a mean of 9.38 days between trauma and surgery. In both groups, four people had a history of old trauma (more than 1-month interval between trauma and surgery), but all other patients had fresh trauma (less than 1 month); there was no significant differences in surgery times between the two groups. In the transconjunctival approach group, 8% of patients (3/38) were female and 92% (35/38) were male. In the subciliary approach group, the prevalence of males (81%, 34/42) was higher than that of females (19%, 8/42). Of all patients with ectropion, nine were male and one was female. Both patients with entropion were men. There was no statistically significant difference between the two groups in the sexual distribution of patients (*p*= 0.12).

The mean age of patients in the transconjunctival group was 29±9 years with a minimum of 17 years and a maximum of 52 years. The mean age of patients in the subciliary group was 34.6±14.2 years with a minimum of 15 years and a maximum of 76 years. All patients with ectropion were between the ages of 17 and 36 years, and patients with entropion were between 20 and 34 years of age. These results indicate that these two complications do not increase based on age.

MVA (41%) and motorcycle accident (33%) were the most common causes of trauma in the patients in this study. Interpersonal violence (15%) and falling from a height (9%) were among other trauma mechanisms. Among patients with ectropion, six cases were caused by an MVA, 3 cases by a motorcycle accident, and one case was caused by interpersonal violence. Among patients with entropion, one case was due to MVA and one case was due to motorcycle accident.

In the transconjunctival (84%, 32/38) and subciliary (95%, 40/42) groups, simple trauma had a higher prevalence than comminuted trauma. In patients with ectropion, eight cases had simple trauma and two cases had comminuted trauma; both patients with entropion had simple trauma. There was no significant difference between the two groups regarding the type of trauma (*p*=0.1).

There were no significant differences between the two groups in terms of the interval between trauma and surgery (*p*= 0.07), history of massage at the site of surgery (*p*= 0.151), history of postoperative epiphora (*p*= 0.059), scleral show (*p*= 0.415), insertion of porex instead of bone during surgery (*p*= 0.91) (Table 1), and the use of suspension sutures during surgery (*p*= 0.057).

**Table1:** Frequency of history of massage, postoperative epiphora, scleral show and insertion of porex

	Type of incision	Total		Presence		Absence		*p* Value
		Percent	Number	Percent	Number	Percent	Number	
History of massage	Transconjunctival	22	58	16	42	38	100	0.151
	Subciliary	30	71	12	29	42	100	
	Total	52	65	28	35	80	100	
Postoperative epiphora	Transconjunctival	29	76	9	24	38	100	0.059
	Subciliary	39	93	3	7	42	100	
	Total	68	58	12	15	80	100	
Scleral show	Transconjunctival	31	82	7	18	38	100	0.415
	Subciliary	37	88	5	12	42	100	
	Total	68	85	12	15	80	100	
Insertion of porex	Transconjunctival	16	42	22	58	38	100	0.091
	Subciliary	25	60	17	40	42	100	
	Total	41	51	39	49	80	100	

Incidence rates of ectropion and entropion in the two studied groups at one month and 5 months after surgery are shown in Table 2 and Table 3, respectively.

**Table2:** Frequency of incidence of ectropion at one month and five months after surgery

Type of incision	Without ectropion		Grade 1		Grade 2		Total		*p* Value
	Percent	Number	Percent	Number	Percent	Number	Percent	Number	
Transconjunctival (1 month after surgery)	87	33	8	3	5	2	100	38	0.482
Subciliary (1 month after surgery)	88	37	2	1	10	4	100	42	
Total (1 month after surgery)	87	70	5	4	8	6	100	80	
Transconjunctival (5 months after surgery)	87	33	8	3	5	2	100	38	0.159
Subciliary (5 months after surgery)	98	41	2	1	0	0	100	42	
Total (5 months after surgery)	92	74	5	4	3	2	100	80	

**Table3:** Frequency of incidence of entropion at one month and five months after surgery

Type of incision	Without ectropion		Grade 1		Grade 2		Total		*p* Value
	Percent	Number	Percent	Number	Percent	Number	Percent	Number	
Transconjunctival (1 month after surgery)	95	36	0	0	5	2	100	38	0.222
Subciliary (1 month after surgery)	100	42	0	0	0	0	100	42	
Total (1 month after surgery)	97	78	0	0	3	2	100	80	
Transconjunctival (5 months after surgery)	94	36	3	1	3	1	100	38	0.322
Subciliary (5 months after surgery)	100	42	0	0	0	0	100	42	
Total (5 months after surgery)	98	78	1	1	1	1	100	80	

The results further indicate that there were no significant differences between the two groups in the frequency of ectropion at one month (*p*= 0.482) and 5 months postoperatively (*p*= 0.159). Moreover, no significant difference in the frequency of entropion between the transconjunctival and subciliary groups were observed one month (*p*= 0.222) and 5 months postoperatively (*p*= 0.322).

## Discussion

The results of this study indicate that trauma involves men more frequently than women, which is similar to the results of previous studies such as those by Champion *et al*. [ 15 ], Clarke *et al*. [ 16 ], and Boilon *et al*. [ 17 ]. The ratio of men to women in this study was 6 to 1, which is comparable to other studies. In surveys conducted in Canada and Australia, this ratio was reported to be 3 to 1 and 2.5 to 1, respectively. The mean age of patients in this study was 31.98±12.31 years with a minimum of 15 and a maximum of 76 years, which is also similar to the results yielded by previous studies. The higher incidence of maxillofacial fractures in the third decade of life may be due to the fact that people in this period of life are more involved than others in sport activities, high risk occupations, or using high-speed vehicles and are more socially active [ 18 ]. In most studies in Iran, such as those by Motamedi [ 19 ] and Ansari [ 20 ] and the present study, car crashes have been reported as the most common cause of fractures.

In the present study, there was no significant difference in the incidence of ectropion and entropion between the transconjunctival and subciliary surgery groups.

Tenzel and Miller [ 21 ] described retroseptal incision. Tessier [ 22 ] introduced preseptal incision. Convers *et al*. [ 23] added lateral canthotomy to the retroseptal incision, which has a better cosmetic advantage than other common methods, because in this method, the scar is behind the lower eyelid. Other advantages of this method are that it is faster and it does not require skin or muscle dissection [ 24 ]. In a similar study by Ridgway *et al*. [ 25], the incidence of ectropion in the subciliary treatment group was 14%, and the incidence of entropion in the transconjunctival group was 1.5%. Their study stated that the appropriate resuspension of the soft of may be a more crucial step in avoiding postoperative complications. The difference between the results of the mentioned study from the present study might be due to the different amounts of edema, different ages of patients, or the use of frost sutures applied postoperatively in only half of the patients. The studies of Ridgway *et al*. [ 25] and Girish *et al*. [ 26] reported that during a three-month follow-up, the incidence of temporary ectropion in the subciliary group was 30%, and the incidence of temporary entropion in the transconjunctival group was 30%. Moreover, they had performed lateral canthotomy in 25 out of 45 patients in transconjunctival group, and this may explain the difference in the results of their study and the current one.

The incidence of scleral show in the transconjunctival group, subciliary group, and all patients was 18%, 12%, and 15%, respectively, in the current study. In the study by Appling *et al*. [ 14 ], the incidence of permanent scleral show was 28% in the subciliary group and 3% in the transconjunctival group. Crosara *et al*. [ 27] compared three surgery methods including subciliary, subtarsal and infraorbital. According to their results, there was no statistically significant difference in ectropion, scleral show, or chronic edema rates among the three groups, which is similar to the current results.

A noteworthy result of the present study was that suspension sutures were used in the transconjunctival approach, and in all but three patients in the subciliary group. Thus, there was no significant difference between the two studied surgical methods in using suspension sutures in preventing ectropion and entropion. This result is similar to that of Bartsich *et al*. [ 28]. The patients who received suspension sutures did not have a higher incidence of complications. In the current study, the use of porex produced no significant difference in the incidence rates of ectropion and entropion in orbital reconstruction.

## Conclusion

In general, the results of this study showed that there is no significant difference between the transconjunctival method and the subciliary method in terms of postoperative complications such as ectropion and entropion. It should be noted, however, that the limited interval time in this study makes it difficult to interpret the results. Therefore, in order to achieve results with more accuracy, it is suggested that the present study be repeated with a larger sample size and in several treatment centers as well as at different time intervals. The results of this study could be the starting point for further studies aimed at selecting the best treatment plan with the fewest complications and improving the quality of life of patients. Finally, based on the results of this study, it can be concluded that the prevalence of complications can be decreased according to the skills of the surgeon and does not have a strict relation with the nature of the procedure.
